# Real-time video analysis allows the identification of large vessel occlusion in patients with suspected stroke: feasibility trial of a “telestroke” pathway in Northwestern Switzerland

**DOI:** 10.3389/fneur.2023.1232401

**Published:** 2023-10-24

**Authors:** Sebastian Thilemann, Christoph Kenan Traenka, Fabian Schaub, Lukas Nussbaum, Leo Bonati, Nils Peters, Joachim Fladt, Christian Nickel, Patrick Hunziker, Marc Luethy, Sabine Schädelin, Axel Ernst, Stefan Engelter, Gian Marco De Marchis, Philippe Lyrer

**Affiliations:** ^1^Department of Neurology and Stroke Center, University Hospital Basel, University of Basel, Basel, Switzerland; ^2^Neurology and Neurorehabilitation, University Department of Geriatric Medicine FELIX PLATTER, University of Basel, Basel, Switzerland; ^3^Department of Emergency, University Hospital Basel, University of Basel, Basel, Switzerland; ^4^Medical Intensive Care Units, University Hospital Basel, Basel, Switzerland; ^5^Anaesthesiology, University Hospital Basel, Switzerland and Emergency Medical Service (EMS) Basel, Basel, Switzerland; ^6^Clinical Trial Unit, University Hospital Basel, University of Basel, Basel, Switzerland; ^7^ICT Service and Support, University Hospital Basel, Basel, Switzerland; ^8^Department of Neurology and Stroke Center, University Hospital St Gallen, St. Gallen, Switzerland

**Keywords:** telestroke, telemedicine, LVO, stroke, pre-hospital, triage, treatment

## Abstract

**Background and aim:**

Loss of time is a major obstacle to efficient stroke treatment. Our telestroke path intends to optimize prehospital triage using a video link connecting ambulance personnel and a stroke physician. The objectives were as follows: (1) To identify patients suffering a stroke and (2) in particular large vessel occlusion (LVO) strokes as candidates for endovascular treatment. We have chosen the Rapid Arterial Occlusion Evaluation (RACE) scale for this purpose.

**Methods:**

This analysis aimed to verify the feasibility of prehospital stroke identification by video assessment. In this prospective telestroke cohort study, we included 97 subjects, in which the RACE score (items: facial palsy, arm and leg motor function, head and gaze deviation, and aphasia or agnosia) was applied, and the assessment videotaped by a trained member of the Emergency Medical Services (EMS) in the field using a mobile device. Each recorded patient video was independently assessed by three experienced stroke physicians from a certified stroke center and compared to the neuroimaging gold standard. Within this feasibility study, the stroke code was not altered by the outcome of the RACE assessment, and all patients underwent the standard procedures within the emergency unit.

**Results:**

We analyzed 97 patients (median age 78 years, 53% women), of whom 51 (52.6%) suffered an acute stroke, 12 (23.5%) of which were due to an LVO and 46 patients had symptoms mimicking a stroke. The sensitivity of stroke identification was 77.8%, and specificity was 53.6%. In regard to the identification of an LVO, sensitivity was 69.4% and specificity was 84.3%. The inter-rater agreement in the RACE-score assessment was ICC = 0.82 (intraclass-correlation coefficient).

**Conclusion:**

These results confirm our hypothesis that the local telestroke concept is feasible. It allows correct (i) stroke and (ii) LVO identification in the majority of the cases and thus has the potential to assist in efficient prehospital triage.

## 1. Introduction

One of today's challenges in stroke medicine is to further decrease the event-to-treatment time. Thus, a lot of effort is invested into shortening and improving every step in the treatment chain. It has been shown in a randomized, blinded, and prospective trial in 2008 that, due to better diagnostic accuracy, the implementation of a telemedical stroke consultation led to a higher thrombolysis rate. However, this was exclusively achieved using a stationary approach at the remote spoke sides (1). As technology is advancing fast, handheld mobile devices could prove themselves useful as pragmatic and easy-to-use tools for the preclinical triage of stroke patients. Consequently, this will enable the acceleration of the prehospital clinical assessment.

We defined telestroke as an application of digitalized telecommunication technology that is video supported, conforms to medical needs, and aligns with standards in acute stroke care; the starting point is the first contact at the site of symptom occurrence. In contrast, the ESO guidelines refer in their current recommendations on telestroke in Europe (2) to the classical definition introduced by Levine and Gorman (3); the most common hub-and-spoke model allows to provide stroke expertise from a telestroke center (TSC, the hub) to remote regional hospitals (spoke).

In order to adapt a local telestroke concept as fast as possible, this feasibility study was conducted in collaboration with the emergency medical service (EMS) Basel and the University Hospital Basel (UHB). The aim is to optimize triage of presumed stroke patients, especially those with large vessel occlusions before arrival in the hospital, using a video link connecting the onsite EMS with the stroke physician at the stroke center on call 24/7. Patients with symptoms suggestive of stroke/LVO could skip the time-consuming ER admission and directly proceed to CT and CTA imaging or directly to the Angiosuite. Consecutively, door-to-needle and/or door-to-groin-puncture-time could potentially be decreased (4, 5), and more patients could receive acute treatment. In contrast to previous studies, our telestroke pathway uses handheld mobile devices with secured connections (6).

Thrombolysis is currently available for ~2–10% of patients globally (7), which could be greatly improved in an optimized setting. Within the UHB catchment area, those numbers ranged from 22% to 29.5% (8). As shown in a systematic review, mass media interventions were capable of raising awareness of stroke and TIA symptoms. However, the expected increase in consecutive ER presentations could not be shown (9). Interestingly, i.v. thrombolysis (IVT) rates still increased after the intervention, which could be related to a campaign effect on healthcare professionals rather than an effect on the targeted population at risk.

Several reasons for prehospital delays have been evaluated and analyzed recently (8). They can be subdivided into unmodifiable risk factors (living alone, younger age, and low baseline NIHSS) and modifiable risk factors (lack of awareness of stroke symptoms, seeing a family doctor before ER admission, and transport other than EMS). The next step in the treatment chain that is to be targeted within the optimization process is directly after the EMS arrival with the patient. In order to detect other possible causes of focal neurological deficits, a first assessment is made by the paramedic before initiation of the telestroke pathway. This includes head trauma, altered mental status due to an infection, metabolic disturbances, or postepileptic symptoms, all of which are commonly referred to as “stroke mimics” (10).

Within the field of EMS, several prehospital stroke scores have been investigated to estimate the likelihood and severity of suspected stroke (11). The identification of LVOs holds especially high potential to improve patient outcomes as 24/7 endovascular thrombectomy (EVT) coordination in stroke centers is a challenging task for stroke centers. Within our rather urban telestroke setting in Northwestern Switzerland, the decision was made to use the Rapid Artery Occlusion Evaluation scale (12), a brief focused assessment, developed to identify LVO in a preclinical setting.

As smartphone devices equipped with high-definition cameras are evolving fast and network coverage is improving rapidly, this technology should be used to further optimize prehospital triage. Stroke patients can be evaluated faster using a handheld device to establish a video connection. However, mobile network coverage is still varying a lot, even in an urban or suburban environment within the UHB catchment area.

The aims of this study are as follows: First, to install a secure real-time video communication between the EMS and the stroke team of the UHB in suspected stroke cases and record the standardized assessment. Second, to evaluate the acquired videos offline with respect to the diagnostic yield of clinical diagnosis of any stroke and large vessel stroke. The subsequent efficacy study, which is running at UHB while this study is being published, adopted these results and evaluates the clinical impact of prehospital triage applying our telestroke path.

## 2. Methods

### 2.1. Patient assessment and video acquisition

Before patients were included in the actual feasibility study, a two-step pilot phase was conducted: (1) A total of 10 healthy volunteers underwent a telestroke assessment within the University Hospital Basel, presenting simulated focal neurological deficits; (2) A total of 10 diagnosed stroke patients, located in UHB‘s Stroke Unit or Neurological ward, were assessed and videotaped. Recordings were later evaluated to optimize the protocol and upcoming EMS training. After this pilot phase, the first patients were included in the UHB emergency department and underwent all procedures described below.

For this study, several preclinical stroke scores were assessed and considered unsuitable for our needs. We sought a brief clinical score that could be obtained fast and be easily supervised by the stroke physician via videoconference. Short transportation times and high EMS staff count were limiting factors regarding total assessment time and complexity (13). We used the RACE, composed of the items: facial palsy, arm and leg motor function, head and gaze deviation, and aphasia or agnosia, counting up to 9 points (12, 14). A RACE score >4P is considered to represent a likely LVO.

In total 20 iPhones 6s^®^ were apportioned to ambulances (10 devices each in day and night shifts alternatively) and EMS staff were trained in three consecutive teaching sessions on 06/2018, 09/2018, and 11/2018. In total, 108 paramedics participated in the interactive teaching courses. In addition, during the first launch of telestroke, short daily sessions were included in the morning briefing. From 09/2018 onwards, paramedic teams could activate the telestroke path 24/7 to get into contact with the stroke physician on duty. A VIDYO^®^-connection was established between a mobile device (iPhone 6s^®^) out-of-hospital and a personal computer, run by the stroke physician, in-hospital. A short briefing on the medical history and relevant medication of the patient was followed by the standardized neurological RACE examination being conducted by the paramedic. The stroke physician supervised the assessment in real time and thus had the opportunity to have parts of the assessment repeated in case of bad video quality or other issues, and no additional assessments ought to be performed. Everything was recorded and saved on an encrypted server. On arrival at UHB, ER standard stroke protocols were applied, according to SOPs, within this feasibility study. No stroke or EVT code was altered.

For the reported prospective cohort study, we aimed to include at least 95 patients according to the power analysis provided by the clinical trial unit (CTU) of the University of Basel. Overall, 97 patients were included between the 5th of May 2018 and the 7th of January 2020.

For analysis of the video, written informed consent was obtained from patients themselves, next of kin, or by a physician not related to this study. After the rollout of telestroke at EMS Basel, no further patients were included within the UHB.

### 2.2. Video evaluation

Each video was randomly assigned to three independent and blinded raters out of a group of stroke physicians. All raters were instructed to watch and assess each video in one sequence. Raters could pause and resume their assigned rating sessions at will. Results, including RACE score, assessment of stroke (y/n), and LVO (y/n) were entered into a secuTrial^®^ eCRF (elektronic Case Report Form) for statistical analysis. Results of these 291 ratings were compared to the neuroimaging gold standard in each patient to evaluate the accuracy of the telestroke assessment in order to identify: (1) Stroke patients out of the population of suspected stroke patients and (2) LVO stroke patients in particular. The presence of a stroke was defined as CTA-proven cerebral vessel occlusion, DWI lesion, or acute intracerebral bleeding. LVO was defined as CCA, ICA, MCA M1/M2, or BA-occlusion in CTA or referring area in perfusion imaging in one case where thrombolysis successfully reopened the occlusion during imaging.

Baseline characteristics of all patients are summarized by diagnosis according to imaging. Categorical data are presented as absolute and relative frequencies and are compared between patients, with and without stroke, using a chi-square test. Numerical values are summarized as median and interquartile, and tested using a Wilcoxon–Mann–Whitney test. The same summaries are provided in stroke patients only by LVO.

The number and proportion of correct assessments of stroke and LVO are listed. The number of patients with 0, 1, 2, and 3 correct telestroke assessments are summarized as absolute and relative frequencies. The number of patients with RACE-score < 5 vs. ≥ 5 is indicated. Furthermore, the sensitivity and specificity of the RACE score are presented in a ROC curve (receiver operating characteristics). Agreement of the RACE scores based on the telestroke videos are quantified using the intra-class correlation (ICC), describing inter-rater agreement. The ICC range is 0–1, with 1 = absolute agreement. The ICC is calculated based on the analysis of variance and is estimated by dividing the variance, which is due to patient-to-patient variability through the total variability seen in the RACE scores. The ICC can have values between 0 and 1. An ICC of 1 would indicate that all variability in the RACE scores can be explained by the difference between patients, and thus, a complete agreement of the raters. All analyses were performed using the R software (version 3.6.2).

Institutional Review Board's (IRB) approval was obtained from Ethikkommission Nordwest- und Zentralschweiz (EKNZ), project-ID 2017-00851.

## 3. Results

Between 5 May 2018 and 7 January 2020, 97 patients with EMS suspected stroke were included in our study. In one patient (TELE-USB-034), no CTA was performed as non-contrast CT imaging revealed a large demarked infarct core already, which due to its size was related to an LVO. In another patient (TELE-USB-089), IVT was administered immediately after the native CT scan and successfully reopened an M1 occlusion displayed in perfusion imaging. Thus, CTA showed the just re-opened vessel instead of an occlusion. In one patient (TELE-USB-010), no cerebral imaging was performed as the treating hospital, which was not the UHB, found a plausible cause for the patient's deficits other than stroke as well as patients TELE-USB-017, −044, −090, and −094, treated at the UHB.

Table 1 displays characteristics, performed cerebral imaging, and administered treatment of all patients, subdivided into the three groups stroke mimics, strokes, and LVO strokes. Compared to mimics, stroke patients of our cohort were more likely to be female (54.9% vs. 50%), were slightly older (median 79 years vs. 77 years), and more severely affected on arrival (median NIHSS score 8.0 vs. 2.0, *p* < 0.001). The characteristics of confirmed stroke patients, according to whether LVO was present, are displayed in Table 1B. LVO patients were more likely to be female patients (66.7 vs. 51.3 %) and presented with a higher NIHSS on admission (median NIHSS score 15.0 vs. 6.0, *p* < 0.001).

**Table 1:** Patient characteristics of all patients according to whether **(A)** stroke or **(B)** LVO was confirmed.

**(A) Confirmed stroke**	**No**	**Yes**	**All**
Number [*n* (%)]	46 (47.4)	51 (52.6)	97 (100)
Female sex [*n* (%)]	23 (50)	28 (54.9)	
Age in years [media*n* (IQR)]	77.0 (65.5, 85.0)	79.0 (69.0, 87,0)	
NIHSS [media*n* (IQR)]	2.0 (0.0, 5.0)	8.0 [5.0, 12.0]	
**Treatment (%)**			
-Both	1 (2.2)	4 (7.8)	5 (5.2)
-EVT	0	4 (7.8)	4 (4.1)
-IVT	2 (4.3)	12 (23.5)	14 (14.4)
-None	43 (93.5)	31 (60.8)	74 (76.3)
**Imaging [*****n*** **(%)]**			
-CT and MRI	19 (41.3)	34 (66.7)	53 (54.6)
-CT only	13 (28.3)	11 (21.6)	24 (24.7)
-MRI only	8 (17.4)	6 (11.8)	14 (14.4)
-None	6 (13)	0	6 (6.2)
**(B) Confirmed LVO**	**No**	**Yes**	**All**
Number [*n* (%)]	39 (76.5)	12 (23.5)	51 (100)
Female sex [*n* (%)]	20 (51.3)	8 (66.7)	
Age in years [media*n* (IQR)]	79.0 (69.0, 87.5)	79.0 (70.0, 81.8)	
NIHSS [media*n* (IQR)]	6.0 (3.5, 10.0)	15.0 [10.8, 21.0]	
**Treatment (%)**			
-Both	0 (0)	4 (33.3)	4 (7.8)
-EVT	1 (2.6)	3 (25.0)	4 (7.8)
-IVT	10 (25.6)	2 (16.7)	12 (23.5)
-None	28 (71.8)	3 (25.0)	31 (60.8)
**Imaging [*****n*** **(%)]**			
-CT and MRI	27 (69.2)	7 (58.3)	34 (66.7)
-CT only	6 (15.4)	5 (41.7)	11 (21.6)
-MRI only	6 (15.4)	0 (0)	6 (11.8)
-None	0 (0)	0 (0)	0 (0)

NIHSS, National Institutes of Health Stroke Scale; EVT, endovascular treatment; IQR, Interquartile range; IVT, intravenous thrombolysis; CT, computer tomography; MRI, magnet resonance imaging.

Of all the 97 patients included, 51 suffered any stroke (52.6%), as confirmed by subsequent brain imaging. For these patients, overall, 153 ratings were performed by three independent raters. In total, 119 out of 153 ratings were correctly attributed to strokes, achieving an overall sensitivity of 77.8%. The other 46 patients without a confirmed stroke were rated correctly as such in 74 out of 138 times, thus achieving an overall specificity of 53.6%. Overall accuracy was 193 out of 291 (66.3%). Likelihood ratios (LR) for stroke were LR+= 1.68 and LR-= 0.41.

Of all patients analyzed, 12 patients suffered an LVO stroke (23.5%). In total, 25 out of 36 assessments correctly identified an LVO stroke with a sensitivity of 69.4% and a specificity of 84.3 %, with 215 out of 255 non-LVO patients identified as such. Accuracy for LVO stroke detection was 240/291 (82.5%). Likelihood ratios for LVO were LR+= 4.43 and LR-= 0.36. Table 2 shows all the diagnostic performance criteria.

**Table 2:** Diagnostic performance criteria.

	**Stroke**	**LVO**
True positive	119	25
True negative	74	215
False positive	64	40
False negative	34	11
Sensitivity	0.778	0.694
Specificity	0.536	0.843
Accuracy	0.663	0.825
Positive likelihood ratio	1.677	4.427
Negative likelihood ratio	0.414	0.362
Positive predictive values	0.650	0.385
Negative predictive values	0.685	0.951

The individual number and proportion of patients with/without confirmed stroke in CTA or MRI suspected as stroke by 0–3 out of 3 raters are displayed in Table 3. Looking closer at patients where an LVO stroke was incorrectly suspected by at least 2 out of 3 raters, multiple factors contributed: 1 out of 3 patients who displayed strong symptoms suffered an ICH, see Table 4 for details. There was a strong correlation between stroke severity and LVO suspicion in both LVO and non-LVO stroke patients. See Table 5 for a detailed description of RACE and LVO assessment. Consider that the tables are on the level of the ratings; thus, each patient is represented three times. The overall inter-rater agreement in the assessment of the RACE score was 0.82 (ICC).

**Table 3:** **(A)** Number and proportion of patients with/without confirmed stroke (via CTA or MRI), suspected “as stroke” by 0–3/3 raters. Within the population of stroke patients, raters positively identified most cases. In patients not suffering a stroke, raters' assessments were widespread. **(B)** Number and proportion of LVOs identified by 0–3 raters. LVO stroke patients were identified by most raters as such, as well as non-LVO patients.

**(A) Number of rater(s) suspecting a stroke**	**n**	**%**
*Confirmed stroke*	Total *n =* 51	100 %
3	33	64.7
2	7	13.7
1	6	11.8
0	5	9.8
*No confirmed stroke*	Total *n =* 46	100
3	12	26.1
2	9	19.6
1	10	21.7
0	15	32.6
**(B) Number of rater(s) suspecting a LVO**	**n**	**%**
*Confirmed LVO*	Total *n =* 12	100
3	5	41.7
2	4	33.3
1	2	16.7
0	1	8.3
*No LVO*	Total *n =* 85	100
3	7	8.2
2	6	7.1
1	7	8.2
0	65	76.5

**Table 4:** Analysis of patients incorrectly suspected to have a LVO stroke by 3 out of 3 and 2 out of 3 raters.

**Patient IDs where 3 out of 3 raters wrongly suspected LVO**	**Diagnosis**	**Patient IDs where 2 out of 3 raters wrongly suspected LVO**	**Diagnosis**
TELE-USB-008	ICH	TELE-USB-019	Prior stroke (RACE 6/9P)
TELE-USB-020	High grade ICA stenosis	TELE-USB-045	Vestibular neuropathy
TELE-USB-030	High-grade ACM Stenosis	TELE-USB-050	Internal capsular stroke
TELE-USB-040	High-grade ICA stenosis	TELE-USB-083	Multiple strokes
TELE-USB-060	Gliobalstoma multiforme WHO IV	TELE-USB-087	Undetermined Stroke
TELE-USB-074	ICH	TELE-USB-095	Stroke of basal ganglia
TELE-USB-079	Post-circulation stroke	

**Table 5:** RACE score distribution of all patients broken down to LVO absence/presence.

**NO confirmed LVO**	**Assessment**	**RACE < 5 [n]**	**RACE ≥5 [n]**	**All [n]**
	YES	12	28	40
	NO	211	4	215
	ALL	223	32	255
**Confirmed LVO**	**Assessment**	**RACE**<**5 [*****n*****]**	**RACE** ≥**5 [*****n*****]**	**All [** ***n*** **]**
	YES	2	23	25
	NO	8	3	11
	ALL	10	26	36

Cutoff values of the RACE were analyzed regarding the prediction of LVO. A RACE > 4 showed a sensitivity of 0.72 and a specificity of 0.87 with an accuracy of 0.86.

## 4. Discussion

As an innovative approach in this feasibility study, our telestroke concept involved the stroke physician earlier in the treatment chain of suspected stroke patients using an encrypted real-time video conference *via* a mobile device. Given the overall performance, our telestroke concept can be considered feasible to identify (i) stroke and (ii) LVO stroke in particular in our patient population. Assessment takes only 2–4 min (median time 194 s) and can be apportioned to avoid unnecessary delay, e.g., in the case of reduced network coverage or incapability of patient collaboration. On the other side, there is the option to have the assessment of RACE items repeated within the telestroke call. This can be considered an advantage compared to simpler alternatives, such as communicating the RACE score only by the paramedics. Furthermore, the visual information gives a clearer picture of the patient's symptoms and thus increases the quality of the assessment (15).

In the majority of patients, a positive selection of stroke patients in general and patients suffering a major stroke due to an LVO could be achieved successfully in this study. For LVO stroke patients in particular, it has been shown before that patient selection using telemedicine, compared to direct admission into the ER, show similar chances of favorable outcome after EVT (16). However, the clinical outcome strongly depends on local conditions. It has recently been tested in a randomized controlled study from Heidelberg, Germany, if direct transfer into the angiosuite would decrease time-to-groin-puncture in EVT patients (17). The study had to be terminated early due to a delay in stroke imaging in the intervention group despite that time from imaging-to-groin puncture could be reduced. Unfortunately, that gain could not be translated into shorter reperfusion time due to increased admission time. In that trial, all patients were clinically assessed by a neurologist using NIHSS. Comparing different telestroke protocols from Europe (18), Australia (19), and the US (20), we learned that the local conditions define certain characteristics. While older publications consider any telemedical approach to stroke medicine, newer publications, such as the ESO 2019 guidelines, refer to any real-time video–audio connection as telestroke. In most settings, a connection between “hub” and “spoke” hospital is established for consultation. In our novel approach, the use of a handheld mobile device on-site allows convenient and flexible initiation and conduction of real-time patient evaluation. This can be considered an extension of the conventional stationary telestroke concept, and is especially valuable in LVO patients as preparation for the interdisciplinary EVT procedure is consuming precious time. The number of EVTs at UHB rose from 38 cases in 2015 to 138 cases in 2020, which reflects the increasing importance of this treatment modality. All investigated stroke scores, developed for the recognition of LVO, hold their specific characteristics and thus vary in complexity and time required (7). RACE was chosen as a quick tool for LVO stroke identification, even in our urban settings. Other scenarios such as telestroke services in the rural Australian outback have different requirements (21). However, when comparing the performance of direct assessment by EMS staff and telemedical evaluation, the latter could be shown to be advantageous (15).

During the process of developing the Basel telestroke protocol, there have been controversies about the acceptance, mainly by older patients, regarding the use of mobile devices for telemedical analysis. However, the COVID-19 pandemic that arose in 2020 changed the perception of non-face-to-face consultations. The current crisis boosted the development of teleconferences in general and telemedicine in particular. Thus, increasing acceptance in emergency settings can be assumed (22). Furthermore, the opportunity to have a digitally supported, prehospital triage might turn out to be an unexpected advantage in the future when new COVID-19 variants or other airborne pathogens spread (23).

In 2 out of 4 patients suffering an ICH, all raters suspected an LVO. It has to be taken into consideration that exclusive clinical discrimination between ischemic and hemorrhagic stroke is, despite great efforts, not possible. All approaches to develop purely clinical scores for pre-imaging recognition of ICH have not been successful enough so far (24). However, prehospital triage of those patients in order to shorten the time to imaging/treatment can be beneficial for those patients as it is proven that ICH patients do benefit from Stroke Unit care and early blood pressure control is critical.

Stroke mimics were only identified by all raters in 1 out of 3 cases, and the specificity of stroke recognition was 53.6%, using the RACE scale in our telemedical approach. However, in 2014, the RACE was published as a simple and quick prehospital tool to predict the presence of LVO in patients with acute stroke (12). The identification of stroke mimics is still a challenge in a preclinical setting (25) as most approaches fail to reach sufficient specificity. Thus, inconsistency in the assessment of mimics using the RACE tool seems reasonable as it was exclusively developed for the identification of LVO strokes. In some patients, despite supervision, RACE was not conducted correctly/completely. Furthermore, prior stroke and symptomatic high-grade stenosis led to an incorrect LVO suspicion; for details see Table 4.

In general, the effect of telestroke intervention could be much stronger in less urban areas (26). Thus, after proving the concept feasible, it might be applied to a wide range of patients allowing efficient transfer coordination to stroke units or stoke centers in Switzerland according to the likelihood of LVO or stroke accordingly (in one of the 10 Swiss stroke centers, which can provide endovascular treatment or otherwise one of the 14 stroke units).

A fully automated evaluation of focal neurological deficits might be a promising approach for the future, but the current technological standards cannot yet be realized. More standardized recording conditions are considered necessary (spacing, background color, and stereo-cameras). First trials allowed vector-based analysis of high-definition video recordings only in a highly standardized laboratory environment. Unfortunately, this cannot be provided in real-life ambulances, and most importantly, it is not feasible to install mounted stereo-cameras. That approach is nevertheless promising for the future of telemedical analysis (27). This represents some of the many challenges to be addressed if we want to adapt technological advances into clinical practice. While political authorities in many countries have already declared a digital health revolution, the legal foundation for widespread implementation of telemedical applications is not yet in sight (23).

Our study has some limitations: (i) Due to technical restrictions, it was not possible to track and analyze every single telestroke contact approach undertaken. We consider technical limitations and workload as the main reasons for failed telestroke execution after interviewing EMS staff and stroke physicians on call. (ii) Only EMS Basel had been involved in the feasibility part of the study. However, more rural areas are considered to have a higher potential for telemedical interventions. (iii) We suffered some technical inconveniences due to a lack of 24/7 technical support. Furthermore, the well-established video conference platform Vidyo^®^ underwent structural changes, resulting in restricted operability during the study, which was challenging to handle in the emergency setting. (iv) Video acquisition and video analysis were not done by the same person within this feasibility study.

Some strengths of our study should be named: (i) The study was conducted under real-world conditions, including obstacles such as reduced network coverage or technical issues, which cannot be solved during an emergency medical situation. This allows us to draw relevant conclusions for real-world situations. (ii) As paramedics of EMS Basel are aware of the time-sensitive nature of stroke in general and intense training has taken place, telestroke path initiation can be considered mostly unbiased. Thus, a representative group of patients was analyzed, which can be confirmed by (a) a portion of LVO strokes out of all strokes and (b) the ratio of strokes to mimics, all roughly representing the numbers we see in the ER. (iii) RACE-score evaluation by the EMS staff has proven to be successfully used in prehospital validation studies (12), as well as in clinical practice (14). Real-time supervision by the stroke physician on call ought to optimize assessment, especially when used for prehospital triage in future.

We can conclude that the Basel telestroke concept is feasible for the identification of LVO and stroke patients in the group of patients with suspected stroke. Further research is warranted to test efficacy in real-time analysis to reduce the time to revascularization treatment in patients with acute ischemic strokes.

## Data availability statement

The raw data supporting the conclusions of this article will be made available by the authors, without undue reservation.

## Ethics statement

The studies involving humans were approved by Ethikkommission Nordwest- und Zentralschweiz (EKNZ), project-ID 2017-00851. The studies were conducted in accordance with the local legislation and institutional requirements. The participants provided their written informed consent to participate in this study.

## Author contributions

ST was responsible for the acquisition of data and wrote the first draft of the manuscript. ST, FS, and SS performed data analysis. All authors made contributions to the study conception and design, reviewed and edited the manuscript, and approved the final version.

## References

[1] Meyer BC Raman R Hemmen T Obler R Zivin JA Rao R. Efficacy of site-independent telemedicine in the STRokE DOC trial: a randomised, blinded, prospective study. Lancet Neurol. (2008) 7:787–95. 10.1016/S1474-4422(08)70171-6 18676180 PMC2744128

[2] Hubert GJ Santo G Vanhooren G Zvan B Campos ST Alasheev A. Recommendations on telestroke in Europe. Eur Stroke J. (2019) 4:101–9. 10.1177/2396987318806718 31259258 PMC6591763

[3] Levine SR Gorman M “Telestroke”: “Telestroke”: the application of telemedicine for stroke. Stroke. (1999) 30:464–9. 10.1161/01.STR.30.2.464 9933289

[4] Mendez B Requena M Aires A Martins N Boned S Rubiera M. Direct transfer to angio-suite to reduce workflow times and increase favorable clinical outcome. Stroke. (2018) 49:2723–7. 10.1161/STROKEAHA.118.021989 30355182

[5] Mendez FM Dajles A Zevallos C Quispe-Orozco D Mendez-Ruiz A Vivanco-Suarez J. Direct transfer to angiosuite triage strategy for patients undergoing mechanical thrombectomy in a rural setting. Stroke. (2021) 1:1–10. 10.1161/SVIN.121.000124 PMC12512333 41584463

[6] Sauser-Zachrison K Shen E Sangha N Ajani Z Neil WP Gould MK. Safe and effective implementation of telestroke in a US community hospital setting. Perm J. (2016) 20:15–217. 10.7812/TPP/15-217 27479951 PMC5101084

[7] Lahr MM Luijckx G-J Vroomen P van der Zee D Buskens E. Proportion of patients treated with thrombolysis in a centralized versus a decentralized acute stroke care setting. Stroke. (2012) 43:1336–40. 10.1161/STROKEAHA.111.641795 22426467

[8] Fladt J Meier N Thilemann S Polymeris A Traenka C Seiffge DJ. Reasons for prehospital delay in acute ischemic stroke. J Am Heart Assoc. (2019) 8:e013101. 10.1161/JAHA.119.013101 31576773 PMC6818040

[9] Lecouturier J Rodgers H Murtagh MJ White M Ford GA Thomson RG. Systematic review of mass media interventions designed to improve public recognition of stroke symptoms, emergency response and early treatment. BMC Public Health. (2010) 10:784–784. 10.1186/1471-2458-10-784 21182777 PMC3022856

[10] Libman RB Wirkowski E Alvir J Rao TH. Conditions that mimic stroke in the emergency department Implications for acute stroke trials. Arch Neurol. (1995) 52:1119–22. 10.1001/archneur.1995.00540350113023 7487564

[11] Vidale S Agostoni E Prehospital stroke scales and large vessel occlusion: a systematic review. Acta Neurol Scand. (2018) 138:24–31. 10.1111/ane.12908 29430622

[12] Perez de la Ossa N Carrera D Gorchs M Querol M Millan M Gomis M. Design and validation of a prehospital stroke scale to predict large arterial occlusion: the rapid arterial occlusion evaluation scale. Stroke. (2014) 45:87–91. 10.1161/STROKEAHA.113.003071 24281224

[13] Nguyen TTM van den Wijngaard IR Bosch J van Belle E van Zwet EW Dofferhoff-Vermeulen T. Comparison of prehospital scales for predicting large anterior vessel occlusion in the ambulance setting. JAMA Neurol. (2021) 78:157–64. 10.1001/jamaneurol.2020.4418 33252631 PMC8015863

[14] Zaidi SF Shawver J Morales AE Salahuddin H Tietjen G Lindstrom D. Stroke care: initial data from a county-based bypass protocol for patients with acute stroke. J Neurointerv Surg. (2017) 9:631–5. 10.1136/neurintsurg-2016-012476 27342763 PMC5520240

[15] Scott IM Manoczki C Swain A Ranjan A McGovern M Tyson A. Prehospital telestroke vs paramedic scores to accurately identify stroke reperfusion candidates: a cluster randomized controlled trial. Neurology. (2022) 99:e2125–36. 10.1212/WNL.0000000000201104 36240100

[16] Moustafa H Barlinn K Prakapenia A Winzer S Gerber J Pallesen L. Endovascular therapy for anterior circulation large vessel occlusion in telestroke. J Telemed Telecare. (2021) 27:159–65. 10.1177/1357633X19867193 31390946

[17] Pfaff JAR Schonenberge S Herweh C Ulfert C Nagel S Ringleh P. Direct transfer to angio-suite versus computed tomography-transit in patients receiving mechanical thrombectomy: a randomized trial. Stroke. (2020) 51:2630–8. 10.1161/STROKEAHA.120.029905 32772684

[18] Martínez-Sánchez P Miralles A de Barros RS Prefasi D Sanz-Cuesta BE Fuentes B. The effect of telestroke systems among neighboring hospitals: more and better? The Madrid telestroke project. J Neurol. (2014) 261:1768–73. 10.1007/s00415-014-7419-3 24957298

[19] Lillicrap T Pinheiro A Miteff F Garcia-Bermejo P Gangadharan S Wellings T. No Evidence of the “weekend effect” in the northern new south wales telestroke network. Front Neurol. (2020) 11:130. 10.3389/fneur.2020.00130 32174885 PMC7057236

[20] Witrick B Zhang D Switzer JA Hess DC Shi L. The association between stroke mortality and time of admission and participation in a telestroke network. J Stroke Cerebrovasc Dis. (2020) 29:104480. 10.1016/j.jstrokecerebrovasdis.2019.104480 31780246 PMC6954319

[21] Prior SJ Reeves NS Campbell SJ. Challenges of delivering evidence-based stroke services for rural areas in Australia. Aust J Rural Health. (2020) 28:15–21. 10.1111/ajr.12579 31990135

[22] Bloem BR Dorsey ER Okun MS. The coronavirus disease 2019 Crisis as catalyst for telemedicine for chronic neurological disorders. JAMA Neurol. (2020) 77:927–8. 10.1001/jamaneurol.2020.1452 32329796

[23] Busti C Gamboni A Calabro G Zampolini M Zedde M Caso V. Telestroke: barriers to the transition. Front Neurol. (2021) 12:689191. 10.3389/fneur.2021.689191 34594291 PMC8476832

[24] Ojaghihaghighi S Vahdati SS Mikaeilpour A Ramouz A. Comparison of neurological clinical manifestation in patients with hemorrhagic and ischemic stroke. World J Emerg Med. (2017) 8:34–8. 10.5847/wjem.j.1920-8642.2017.01.006 28123618 PMC5263033

[25] Lumley HA Flynn D Shaw L McClelland G Ford G White P. A scoping review of pre-hospital technology to assist ambulance personnel with patient diagnosis or stratification during the emergency assessment of suspected stroke. BMC Emerg Med. (2020) 20:30. 10.1186/s12873-020-00323-0 32336270 PMC7183583

[26] Lazarus G Permana AP Nugroho SW Audrey J Wijaya DN Widyahening IS. Telestroke strategies to enhance acute stroke management in rural settings: a systematic review and meta-analysis. Brain Behav. (2020) 10:e01787. 10.1002/brb3.1787 32812380 PMC7559631

[27] Ali F Hamid U Zaidat O Bhatti D Kalia JS. Role of artificial intelligence in telestroke: an overview. Front Neurol. (2020) 11:559322. 10.3389/fneur.2020.559322 33117259 PMC7576935

